# Artificial Intelligence integration and health system performance: effects on diagnostic accuracy, operational efficiency, and workforce outcomes in medical imaging departments

**DOI:** 10.3389/fpubh.2026.1845439

**Published:** 2026-07-13

**Authors:** Abdulaziz S. Aljibali

**Affiliations:** Department of Radiology, College of Medicine, Qassim University, Buraidah, Al-Qassim, Saudi Arabia

**Keywords:** Artificial Intelligence, computed tomography, diagnostic accuracy, medical imaging, technology adoption

## Abstract

**Background:**

The rapid digitalization of healthcare has positioned Artificial Intelligence (AI) as a key driver of transformation in medical imaging. While its technical capabilities are well established, evidence on its real-world impact on departmental performance and workforce dynamics remains limited.

**Objective:**

This study evaluated the impact of AI integration on diagnostic accuracy, operational efficiency, and staff performance in medical imaging departments.

**Methods:**

A quantitative cross-sectional design was employed using a structured, validated Likert-scale questionnaire administered to healthcare professionals (*N* = 400), including radiologists, technologists, and department managers. Data were analyzed using descriptive statistics, Pearson correlation, and multiple linear regression to assess the contribution of AI-related factors to departmental performance.

**Results:**

AI integration explained 57.8% of the variance in departmental performance (*R*^2^ = 0.578, *p* < 0.001). Diagnostic accuracy (*β* = 0.236, *p* < 0.001) and operational efficiency (*β* = 0.306, *p* < 0.001) emerged as statistically significant positive predictors of departmental performance, with operational efficiency demonstrating the strongest effect. Although Staff Performance demonstrated a positive association with departmental performance, it did not remain a statistically significant independent predictor after multivariate adjustment.

**Conclusion:**

AI integration improves both clinical effectiveness and operational performance in medical imaging departments. Effective implementation requires workflow integration, workforce training, and alignment with broader health system priorities. Future research should explore longitudinal and multi-center impacts on patient and population health outcomes.

## 1 Introduction

Artificial Intelligence (AI) has emerged as a transformative force in healthcare, fundamentally redefining the landscape of diagnostic imaging (1, 2). Early applications in radiology were largely limited to rule-based computer-aided detection systems; however, the advent of advanced deep learning architectures has enabled highly sophisticated capabilities, including automated image segmentation, anomaly detection, predictive modeling, and intelligent workflow prioritization (3–6). These advances have led to substantial improvements in diagnostic accuracy, with multiple studies demonstrating performance levels comparable to, or in some cases surpassing, those of expert radiologists under controlled experimental conditions (6, 7).

Despite these promising developments, the translation of AI from controlled validation settings into real-world clinical environments remains insufficiently characterized (8). Most existing studies emphasize algorithmic performance metrics, often overlooking the broader operational implications of AI deployment within healthcare systems (9, 10). In practice, medical imaging departments operate under increasing pressure to manage rising patient volumes, reduce reporting turnaround times, and address workforce shortages and burnout among radiologists (11–14). In this context, AI should not be evaluated solely as a diagnostic adjunct but as an integrated system capable of enhancing both clinical decision-making and departmental efficiency.

Within medical imaging practice, AI applications increasingly include automated image triage systems, AI-assisted lesion detection algorithms for radiography and computed tomography (CT), workflow prioritization software, speech recognition-assisted reporting systems, and automated image segmentation tools integrated within Picture Archiving and Communication Systems (PACS). These technologies are designed not only to support diagnostic interpretation but also to improve workflow coordination and departmental efficiency.

The integration of AI-driven technologies aligns closely with the strategic priorities of Saudi Vision 2030, which emphasizes digital transformation, healthcare optimization, and sustainable service delivery (15–17). While significant investments have been made to modernize healthcare infrastructure across Saudi Arabia, empirical evaluations of AI implementation remain disproportionately concentrated in major metropolitan centers and often focus on isolated technical outcomes (16, 18). This creates a critical evidence gap regarding the practical impact of AI in emerging and semi-urban healthcare regions, such as the Qassim Region, where healthcare systems face distinct challenges including variable caseloads, limited specialist availability, and resource constraints.

A further limitation in the current body of literature is the insufficient consideration of human and organizational factors influencing AI adoption (19). The Technology Acceptance Model provides a well-established framework for understanding how perceived usefulness and ease of use shape user acceptance and subsequent system performance. Within radiology, the perception of AI as either a supportive clinical tool or a disruptive threat can significantly influence its effectiveness. When appropriately integrated, AI has the potential to improve not only diagnostic accuracy but also workflow efficiency, decision confidence, and job satisfaction among healthcare professionals (20–22). Conversely, inadequate integration strategies may hinder adoption and limit the realization of AI’s full benefits (23–26).

To address these gaps, this study presents a comprehensive, multidimensional evaluation of AI implementation within medical imaging departments in the Qassim Region. Specifically, it investigates the combined impact of AI-driven diagnostic support, workflow automation, and workforce enablement on overall departmental performance. By shifting the analytical focus from isolated algorithmic validation to real-world clinical and operational outcomes, this research provides context-specific evidence to inform policy development, optimize imaging workflows, and support the scalable integration of AI technologies in regional healthcare systems.

Despite the growing body of literature on Artificial Intelligence in healthcare, most existing studies have concentrated on algorithm development, technical validation, and diagnostic accuracy under controlled experimental conditions. Comparatively little attention has been given to understanding how AI integration influences broader organizational outcomes, including operational efficiency, workforce performance, and overall departmental effectiveness within routine clinical practice. Furthermore, evidence from emerging healthcare regions remains limited, particularly within the context of Saudi Arabia’s ongoing healthcare transformation under Vision 2030. The present study was therefore undertaken to address this gap by providing a comprehensive assessment of the clinical, operational, and workforce-related effects of AI implementation in medical imaging departments. Given the rapid expansion of AI technologies in healthcare and increasing demands for efficient, high-quality diagnostic services, this investigation is both timely and relevant for informing policy, management decisions, and future digital health strategies.

## 2 Methodology

### 2.1 Research design and framework

**Figure 1:**
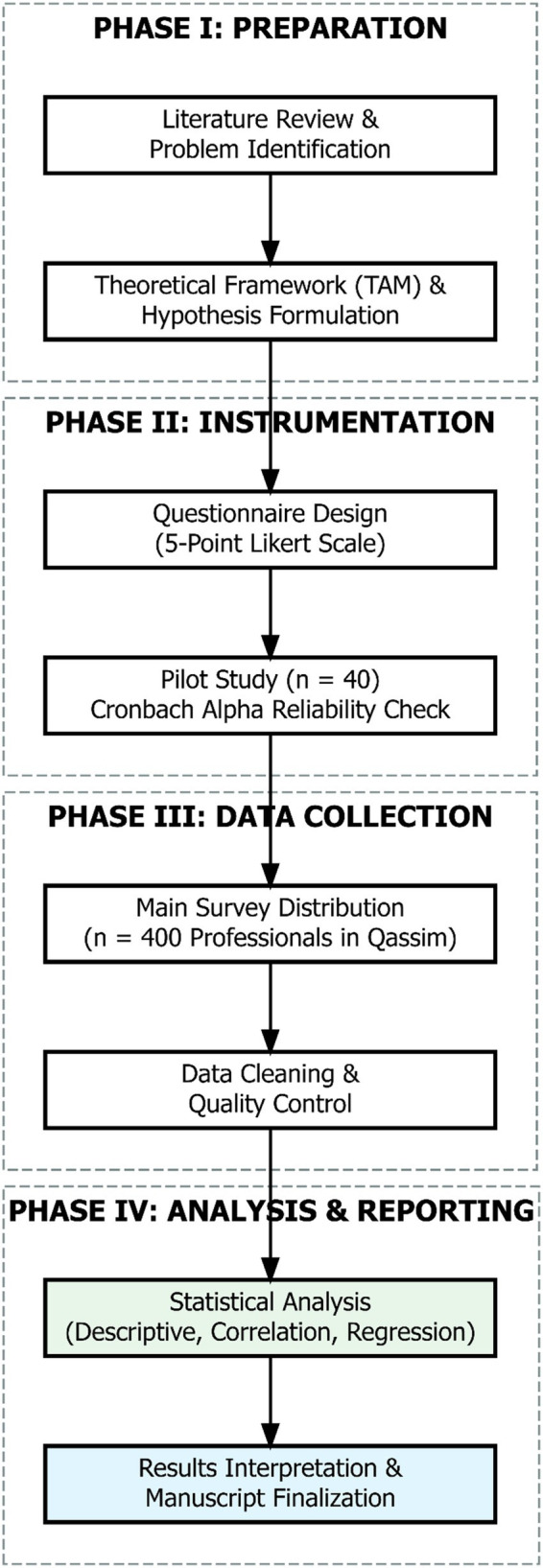
Methodological workflow for evaluating AI integration outcomes.

This study employed a cross-sectional, quantitative research design to evaluate the systemic impact of Artificial Intelligence (AI) on medical imaging departments (27). The conceptual framework is grounded in the Technology Acceptance Model (TAM), extending the constructs of “Perceived Usefulness” and “Perceived Ease of Use” into measurable clinical outcomes: diagnostic accuracy (IV1), operational efficiency (IV2), and staff performance/job satisfaction (IV3). This multi-dimensional approach allows for a holistic assessment of departmental performance (DV) beyond mere algorithmic validation as depicted in Figure 1.

### 2.2 Setting and participants

The study was conducted within the medical imaging departments of the Qassim Region, Saudi Arabia. Participants were recruited from a combination of public hospitals, private healthcare institutions, tertiary referral centers, and diagnostic imaging facilities across the Qassim Region. Recruitment was conducted through institutional electronic survey distribution, departmental communication channels, and direct professional outreach targeting imaging personnel involved in AI-assisted clinical workflows. This region provides a critical representative sample of the Kingdom’s healthcare transformation under Vision 2030. A non-probability purposive sampling technique was utilized to target healthcare professionals, including radiologists, radiographers, imaging technicians, and department managers, who interact with AI-integrated systems in their daily clinical workflow.

Sample size determination was performed using G*Power version 3.1 based on the recommendations of Cohen for multiple linear regression analysis. Assuming a medium effect size (*f*^2^ = 0.15), a significance level of *α* = 0.05, statistical power of 0.95, and three predictor variables, the minimum required sample size was calculated to be 119 participants. This calculation was based on Cohen’s formula for multiple regression and is consistent with established recommendations for behavioral and health sciences research. The final sample size of 400 participants substantially exceeded the minimum requirement, thereby enhancing statistical precision, reducing sampling error, and increasing the robustness and generalizability of the findings (28).

### 2.3 Instrumentation and pilot testing

Data was gathered using a structured, self-administered questionnaire consisting of 25 items across five sections, measured on a 5-point Likert scale (1 = Strongly Disagree to 5 = Strongly Agree).

- Content validity: the instrument underwent a formal review by an expert panel (*n* = 3) specializing in health informatics and radiology to ensure the items accurately captured the technical and operational nuances of AI adoption.
- Pilot study: a pilot test was conducted with 40 imaging professionals to assess the instrument’s internal consistency. Cronbach’s Alpha coefficients for all constructs ranged from 0.82 to 0.91, exceeding the 0.70 threshold for high reliability.

### 2.4 Data collection procedure

The survey was distributed electronically and in person between January 2025 and January 2026. Participation was voluntary, and anonymity was strictly maintained to mitigate social desirability bias. Ethical clearance was obtained from the institutional review board, and informed consent was secured from all participants prior to data entry.

### 2.5 Ethical considerations

The study was conducted in strict accordance with the ethical principles outlined in the Declaration of Helsinki. Prior to data collection, Ethical approval was obtained from the Committee of Research Ethics, Deanship of Graduate Studies and Scientific Research, Qassim University (Approval No. 26.6.6).

To ensure the highest standards of participant protection, the following protocols were implemented:

- *Informed consent*: all participants were provided with a plain-language explanatory statement detailing the study’s objectives, the voluntary nature of participation, and their right to withdraw at any stage without prejudice. Digital or written informed consent was mandatory prior to accessing the survey instrument.
- *Anonymity and confidentiality:* To mitigate potential social desirability or professional bias, a double-blind data collection protocol was employed. No personally identifiable information (PII), such as names, employee IDs, or specific hospital branch names, was collected. Data were aggregated at the regional level to ensure that individual responses could not be traced back to specific personnel or imaging units.
- *Data security*: All raw digital data were encrypted and stored on a secure, password-protected server accessible only to the primary research team. In compliance with data retention policies, all primary data will be permanently deleted following the mandatory period of 5 years post-publication.

### 2.6 Statistical analysis

Data analysis was performed using R (v.4.3.2) and SPSS (v.28.0). The analytical hierarchy was structured as follows: descriptive statistics: calculation of means, standard deviations, and frequency distributions to profile the sample and AI impact scores. Bivariate analysis: Pearson correlation coefficients were computed to examine the strength and direction of the linear relationships between the IVs and the DV. Multivariate analysis: a multiple linear regression model was constructed to determine the predictive weight (*β*) of each AI factor on departmental performance, while controlling for potential multicollinearity via variance inflation factor (VIF) testing.

## 3 Results

### 3.1 Sample characteristics

The demographic and professional characteristics of the study participants (*N* = 400) are presented in Table 1. The sample comprised a predominantly female workforce (62.0%) and reflected the multicultural composition of the healthcare sector, with non-Saudi professionals accounting for 57.0% of respondents. The age distribution indicated a relatively experienced workforce, with approximately two-thirds of participants (67.0%) aged 35 years or older.

**Table 1:** Demographic and professional profile of medical imaging personnel (*N* = 400).

Category	Characteristic	*n*	(%)
Gender	Male	152	38
	Female	248	62
Nationality	Saudi	172	43
	Non-Saudi	228	57
Age group	Below 25 years	76	19
	25–34 years	56	14
	35–44 years	116	29
	45–54 years	72	18
	55 years and above	80	20
Professional role	Radiologist	68	17
	Radiographer/technologist	104	26
	Imaging technician	104	26
	Dept. supervisor/manager	124	31
Years of experience	Less than 5 years	92	23
	5–10 years	84	21
	11–15 years	144	36
	More than 15 years	80	20

From an organizational perspective, the sample included representation from both operational and leadership roles, as 31.0% of respondents held managerial or supervisory positions. In addition, a substantial proportion of participants (56.0%) reported more than 10 years of professional experience in clinical practice. This distribution suggests that the responses were informed by individuals with considerable practical exposure to diagnostic imaging workflows and organizational processes.

### 3.2 Bivariate correlation and network analysis

**Figure 2:**
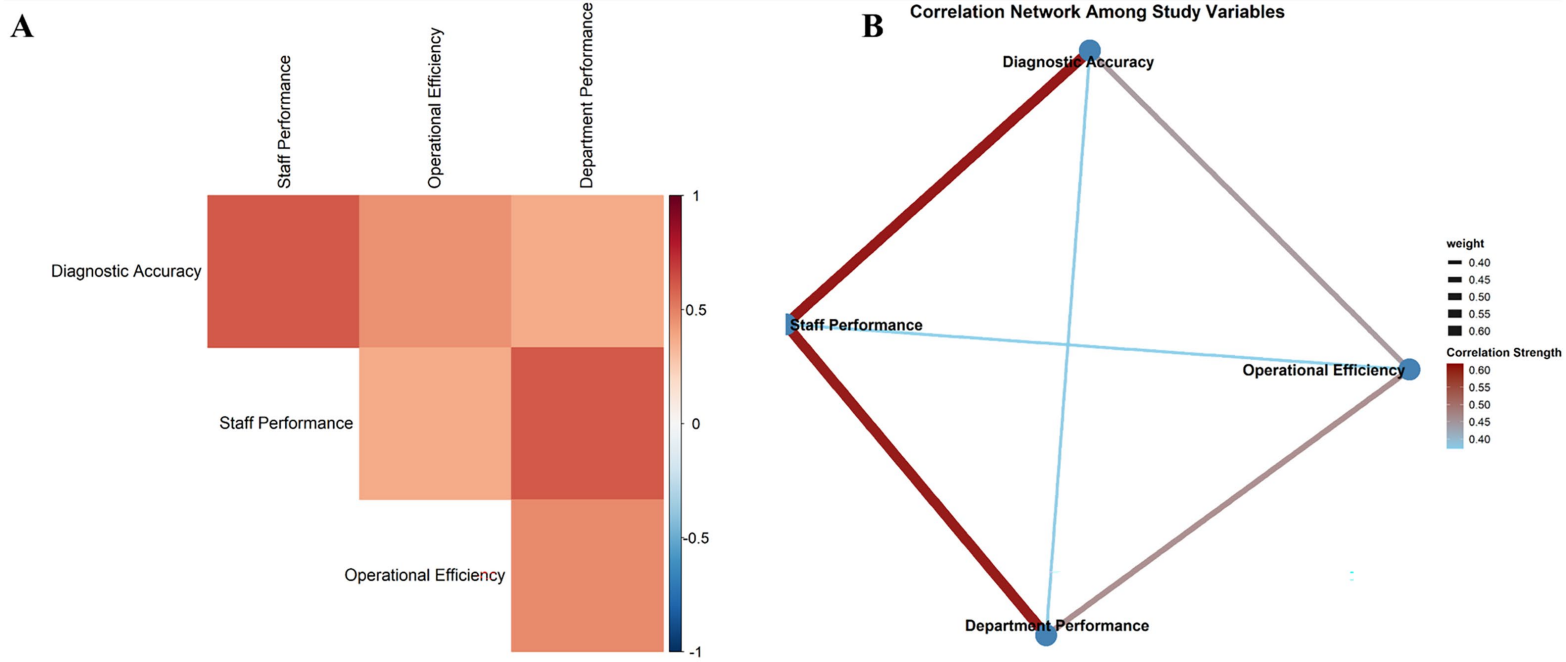
Visualization of the bivariate relationships among AI-integration constructs. **(A)** A lower-triangle heatmap depicting the Pearson correlation matrix, where darker red shading indicates stronger positive correlations. **(B)** A topological correlation network plot illustrating the interdependencies among study variables; edge thickness and color intensity correspond to the strength of the correlational pathways.

To visualize the interdependencies among the primary study constructs, diagnostic accuracy, staff performance, operational efficiency, and department performance, a dual-panel correlation analysis was conducted (Figure 2).

A lower-triangle correlation heatmap, as depicted in Figure 2A, shows the bivariate relationships across all variables. The color gradient, ranging from cool blue (negative correlation) to dark red (positive correlation), indicates that all investigated factors exhibit significant positive associations. The most pronounced relationships, denoted by the darkest red shading, are observed between staff performance and diagnostic accuracy, and between staff performance and department performance. Figure 2B further contextualizes these relationships through a topological correlation network analysis. In this visualization, the nodes represent the core constructs, while the edges (connecting lines) map the strength of the correlations. The edge weight and color intensity corroborate the findings from the heatmap: the thickest, darkest red pathways form a distinct triad connecting staff performance to both diagnostic accuracy and department performance. Conversely, the lighter, thinner edges (such as the connection between diagnostic accuracy and department performance) indicate comparatively weaker, though still positive, associations.

### 3.3 Analysis of regression coefficients

**Figure 3:**
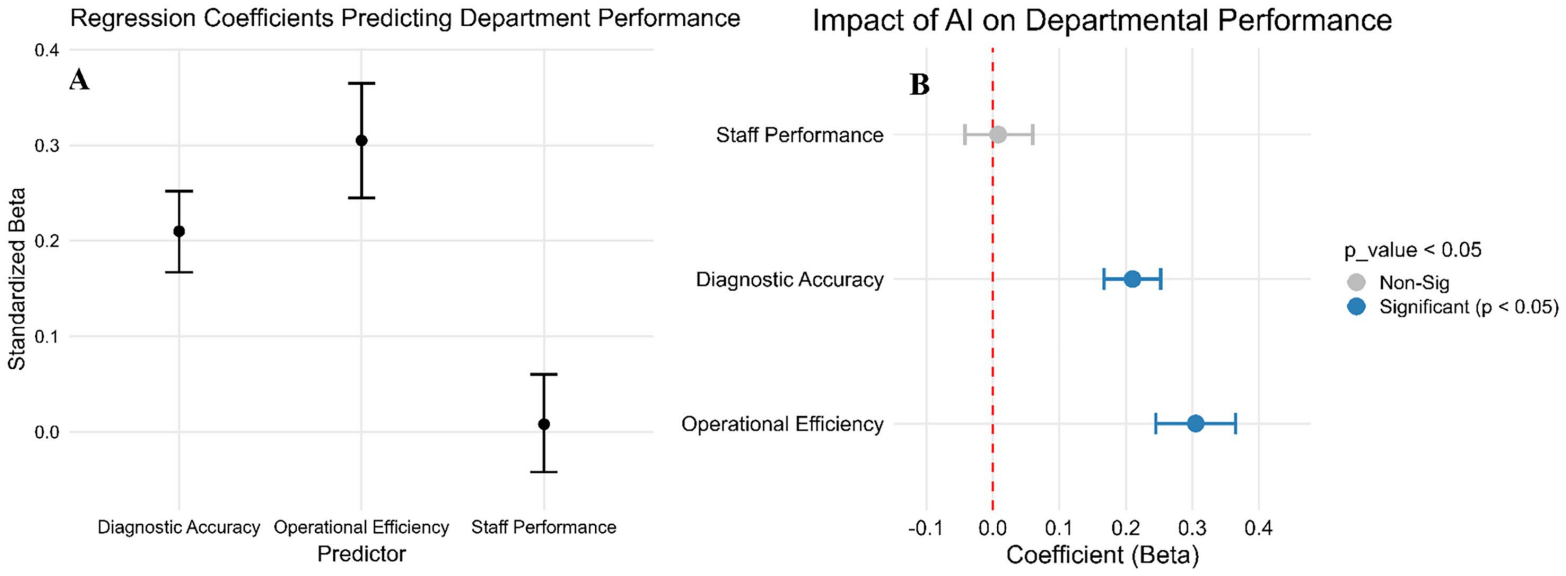
Visual representation of the multiple linear regression model predicting departmental performance. **(A)** A coefficient plot illustrating the standardized beta values and error margins for diagnostic accuracy, operational efficiency, and staff performance. **(B)** A forest plot displaying the coefficients relative to the null effect line (red dashed line). Variables with confidence intervals that do not cross zero are marked in blue, indicating statistical significance (*p* < 0.05), while the non-significant predictor is marked in grey.

To further elucidate the predictive power of the independent variables on departmental performance, the results of the multiple linear regression model were visualized using coefficient and forest plots (Figure 3). Operational efficiency exhibits the most substantial positive predictive weight, positioning it as the primary driver of departmental success. Diagnostic accuracy follows as the second strongest predictor, demonstrating a distinct positive impact. In contrast, the standardized beta for staff performance is situated near zero, with confidence intervals closely centered around the null effect estimate (Figure 3A). While, Figure 3B reinforces this hierarchy through a horizontally oriented forest plot mapping the coefficients alongside their confidence intervals. The vertical red dashed line represents the null hypothesis (a coefficient of zero). The analysis visually confirms that both operational efficiency and diagnostic accuracy are statistically significant predictors (*p* < 0.05), as their confidence intervals (highlighted in blue) fall entirely to the right of the zero line. Conversely, the confidence interval for Staff Performance (highlighted in grey) intersects the zero line, visually denoting it as a non-significant independent predictor within this specific multivariate framework.

### 3.4 Model performance and variance explained by the regression model

**Figure 4:**
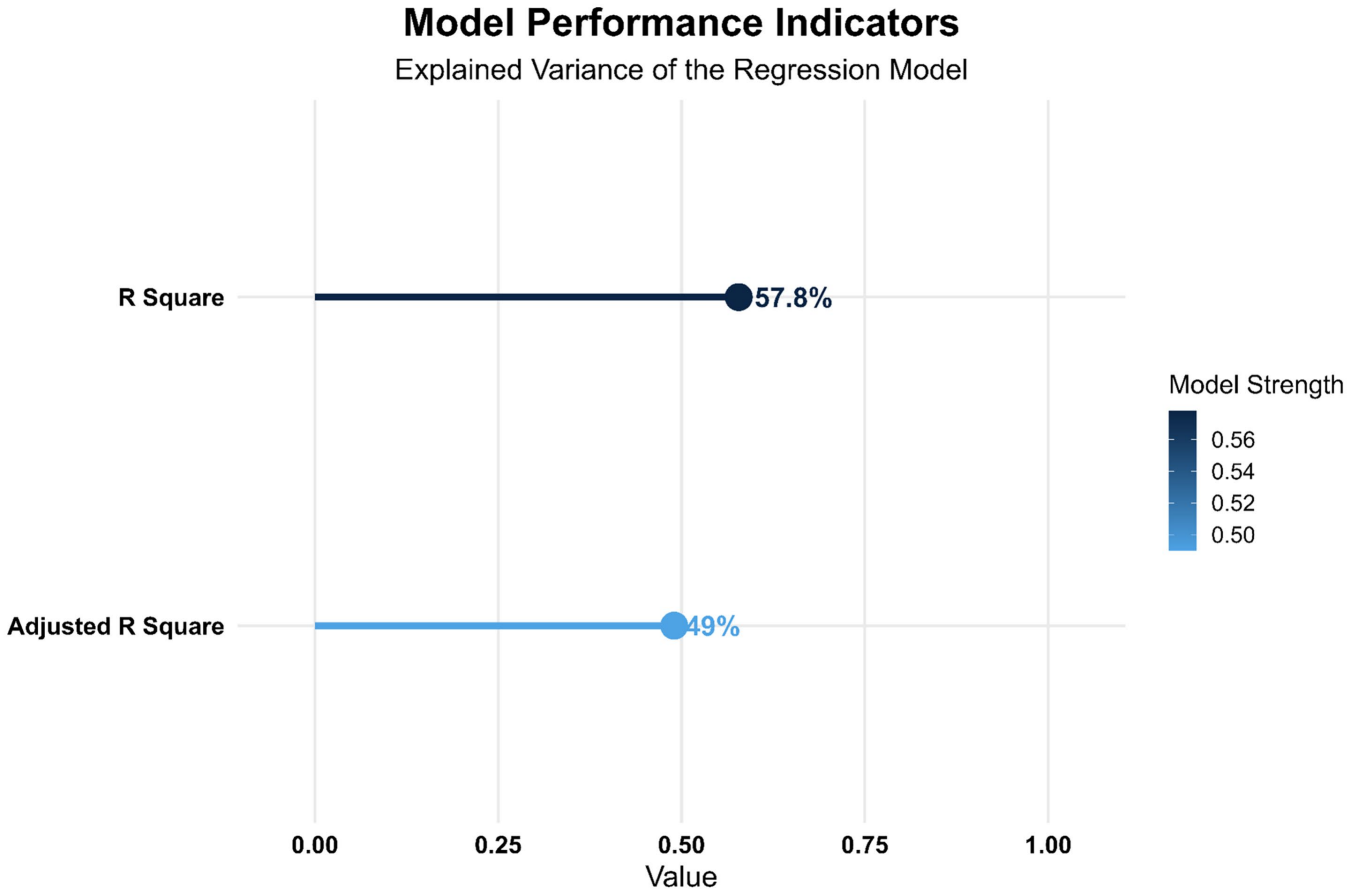
Model performance indicators and partitioning of variance in the regression model. Model performance metrics showing the coefficient of determination (*R*^2^ = 0.578) and adjusted coefficient of determination (Adjusted *R*^2^ = 0.490), indicating the proportion of variance in departmental performance explained by the independent variables after accounting for model complexity. The regression model explained 57.8% of the variance in departmental performance, while 42.2% remained unexplained.

The regression model demonstrated moderate explanatory power, accounting for 57.8% of the variance in departmental performance outcomes (*R*^2^ = 0.578). After adjusting for the number of predictors and model complexity, the Adjusted *R*^2^ value was 0.490, indicating that the model retained moderate explanatory power after correction for potential model overfitting (Figure 4).

### 3.5 AI impact profile

**Figure 5:**
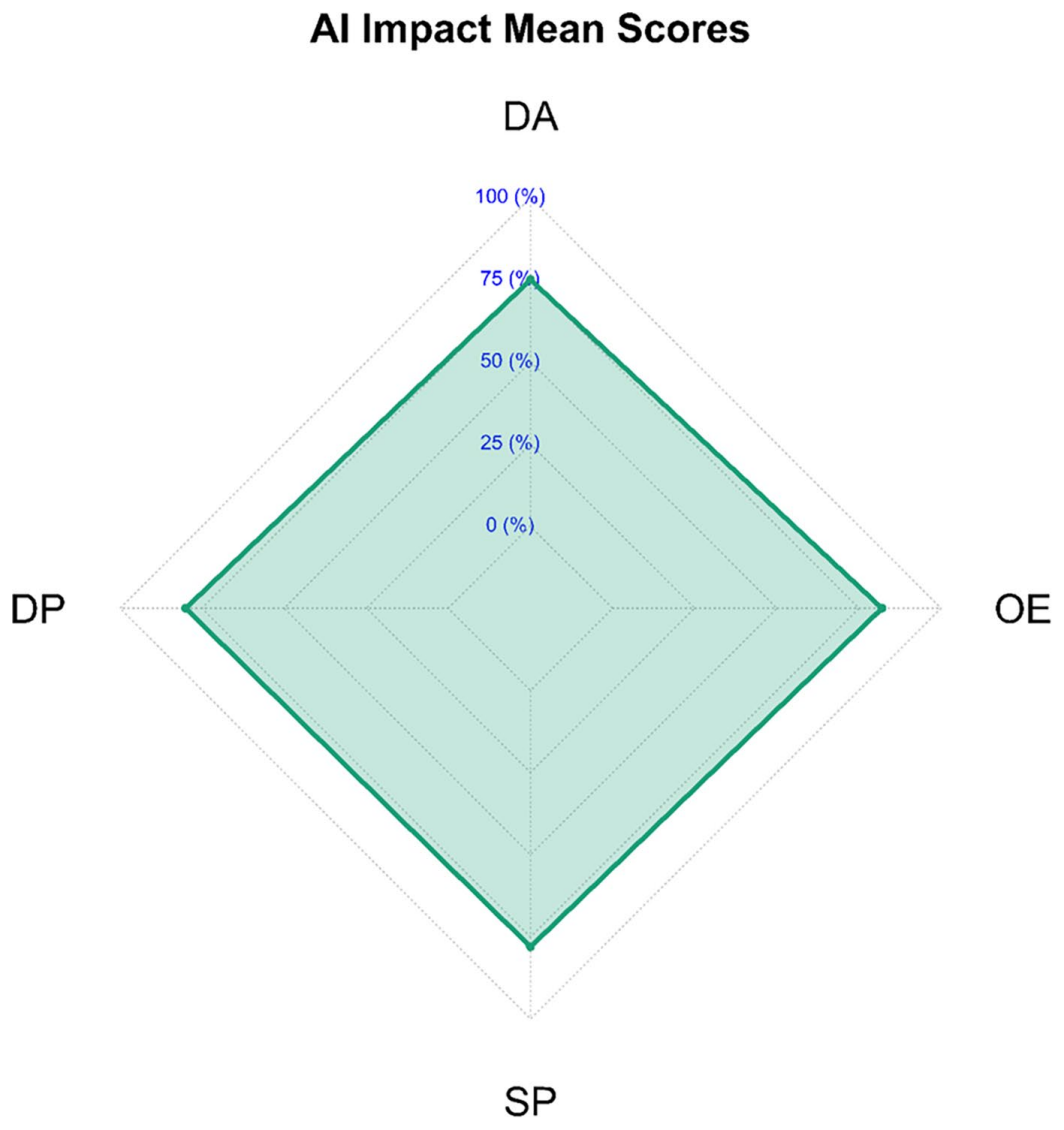
Radar chart illustrating the multivariate profile of perceived AI impact. The nodes map the normalized mean scores across the four primary study constructs (diagnostic accuracy, operational efficiency, staff performance, and departmental performance), reflecting a consistently high and symmetrical perceived benefit across all evaluated dimensions.

To graphically represent the central tendencies of the primary study constructs, a multivariate radar chart was generated mapping the perceived AI impact scores (Figure 5). The chart illustrates the normalized mean scores, expressed as percentages, for diagnostic accuracy (DA), operational efficiency (OE), staff performance (SP), and departmental performance (DP). The resulting polygon demonstrates a highly symmetrical distribution, with the mean scores for all four constructs clustering at the 75% impact threshold. This uniform expansion from the center indicates a strong consensus among the 400 respondents: AI is not perceived merely as a specialized tool for isolated tasks, but rather as a balanced, systemic intervention that exerts an equally robust and positive influence across both technical diagnostic dimensions (DA, OE) and human-organizational outcomes (SP, DP).

### 3.6 Hypothesis testing

In the current study, two of the three research hypotheses were empirically supported (Table 2).

**Table 2:** Summary of hypothesis testing results.

Hypothesis	Path/relationship	*β* coefficient	*p*-value	Decision
H1	Improvements in diagnostic accuracy resulting from AI integration positively influence departmental performance in medical imaging departments.	0.236	<0.001	Supported
H2	Improvements in operational efficiency resulting from AI integration positively influence departmental performance in medical imaging departments.	0.306	<0.001	Supported
H3	Improvements in staff performance and job satisfaction resulting from AI integration positively influence departmental performance in medical imaging departments.	—	>0.05	Not supported

Based on the conceptual framework and prior evidence regarding AI adoption in healthcare, the following hypotheses were formulated:

H1: Improvements in diagnostic accuracy resulting from AI integration positively influence departmental performance in medical imaging departments.

H2: Improvements in operational efficiency resulting from AI integration positively influence departmental performance in medical imaging departments.

H3: Improvements in staff performance and job satisfaction resulting from AI integration positively influence departmental performance in medical imaging departments.

H1: Diagnostic accuracy → departmental performance. Diagnostic accuracy demonstrated a statistically significant positive association with departmental performance (*β* = 0.236, *p* < 0.001), indicating that improvements in diagnostic reliability contribute meaningfully to organizational outcomes. Therefore, Hypothesis 1 was supported.

H2: Operational efficiency → departmental performance. Operational efficiency emerged as the strongest predictor in the regression model (*β* = 0.306, *p* < 0.001), highlighting the central role of workflow optimization and process management in enhancing departmental productivity and service delivery. Accordingly, Hypothesis 2 was supported.

H3: Staff performance → departmental performance. Although staff performance demonstrated a positive association with departmental performance, its regression coefficient did not remain statistically significant after controlling for the other predictors in the multivariate model, as reflected by the confidence interval crossing the null value. This finding suggests that staff-related outcomes may contribute indirectly to departmental performance through operational and workflow-related mechanisms enabled by AI technologies. Therefore, Hypothesis 3 was not supported in the final regression model.

Model diagnostics further confirmed the stability and reliability of the regression estimates. The standard error of the estimate was relatively low (0.371), indicating good model fit. In addition, variance inflation factor (VIF) values for all predictors were below the commonly accepted threshold of 3, suggesting that multicollinearity was not a concern in the analysis.

## 4 Discussion

This study provides an empirical evaluation of Artificial Intelligence (AI) integration within medical imaging departments in the Qassim Region of Saudi Arabia. As the Kingdom advances its Health Sector Transformation Program under Vision 2030, digital health technologies are increasingly embedded within routine clinical workflows rather than functioning as optional adjunct tools (29). In the present study, the multivariate analysis demonstrates that AI-related improvements in diagnostic accuracy, operational efficiency, and staff performance collectively explain a substantial proportion of variance in departmental performance (*R*^2^ = 0.578, *p* < 0.001). From a public health systems perspective, this level of explanatory power indicates that AI implementation is not limited to technical enhancement of diagnostic tools but represents a structural component of service delivery modernization. These findings suggest that AI-enabled workflows can support the transition from volume-driven service provision toward value-based healthcare models, which emphasize quality, efficiency, and patient-centered outcomes. Such alignment is consistent with national priorities aimed at improving service accessibility, optimizing resource utilization, and strengthening health system resilience in response to demographic growth and increasing demand for diagnostic imaging services.

### 4.1 The role of diagnostic accuracy in patient safety and clinical decision-making

The current study also confirms that AI technologies were perceived to improve diagnostic accuracy, which in turn contributes to overall departmental performance (*β* = 0.236, *p* < 0.001). These findings reinforce the clinical decision-support paradigm in which AI systems augment radiologists’ interpretive capabilities by assisting in pattern recognition, anomaly detection, and prioritization of high-risk cases. Rather than replacing clinical expertise, AI appears to function as an enabling technology that enhances diagnostic reliability and consistency.

The positive association between diagnostic accuracy and staff performance (*r* = 0.619) further suggests that reliable AI-assisted outputs may strengthen clinician confidence and reduce cognitive workload associated with high-volume image interpretation. This relationship has direct implications for patient safety, as improved diagnostic precision reduces the likelihood of delayed or missed diagnoses, particularly in time-sensitive clinical areas such as oncology, stroke management, and trauma care.

From a health equity perspective, AI-enabled diagnostic support may also reduce disparities in service quality between urban and peripheral healthcare facilities. In regional healthcare networks where specialized radiology expertise may be unevenly distributed, standardized AI-assisted analysis can contribute to more consistent diagnostic outcomes across facilities. This capability is particularly relevant in decentralized healthcare systems seeking to maintain uniform standards of care across geographically dispersed populations.

### 4.2 Operational efficiency as a determinant of health system sustainability

Among the predictors examined, operational efficiency emerged as the strongest independent determinant of departmental performance (*β* = 0.306, *p* < 0.001). This finding underscores the importance of workflow optimization as a central mechanism through which AI delivers measurable organizational benefits. Operational efficiency in this context encompasses reductions in report turnaround time, automation of routine administrative tasks, improved scheduling coordination, and more effective utilization of diagnostic equipment.

These improvements have important implications for health system sustainability. Enhanced efficiency enables imaging departments to accommodate increasing diagnostic demand without proportional expansion of workforce capacity or infrastructure investment. In resource-intensive environments where advanced imaging equipment represents significant capital expenditure, maximizing utilization rates is essential for maintaining financial sustainability and service continuity.

Furthermore, improved workflow efficiency may indirectly influence broader healthcare system outcomes, including reduced patient waiting times, shorter hospital length of stay, and improved patient throughput. Collectively, these operational gains contribute to improved system responsiveness and support strategic objectives related to cost containment, service quality, and long-term healthcare system resilience. The prominent role of operational efficiency observed in our findings (*β* = 0.306) aligns with recent systematic evaluations of radiological workflows, which demonstrate that AI integration can reduce median reporting times by up to 36% and turnaround times by nearly an hour, particularly in emergent cross-sectional imaging (30).

### 4.3 Staff performance and the human-in-the-loop model of AI implementation

Our study also focused on the human dimension of AI adoption, specifically the relationship between AI utilization and staff performance. While staff performance demonstrated a strong positive correlation with departmental outcomes (*r* = 0.617), it did not remain a statistically significant independent predictor after adjustment for the other variables in the regression model. This pattern suggests that the impact of staff-related factors on organizational performance may be partially mediated through improvements in workflow processes enabled by AI technologies.

Importantly, the findings provide empirical support for the human-in-the-loop model of AI implementation, in which human expertise remains central to clinical decision-making while automated systems support efficiency and accuracy. Rather than diminishing professional roles, AI appears to facilitate role transformation by reducing repetitive or routine tasks and allowing clinicians to focus on higher-value activities such as clinical consultation, interdisciplinary collaboration, and patient communication.

From a workforce sustainability perspective, these changes may contribute to improved job satisfaction and reduced occupational burnout, both of which are critical concerns in diagnostic imaging professions globally. Our empirical support for the human-in-the-loop model corroborates recent frameworks of ‘diagnostic complementarity,’ which posit that human–AI collaboration yields performance superior to either component alone. Rather than substituting clinical expertise, AI offloads routine cognitive tasks, thereby reducing automation anxiety and occupational burnout while allowing radiologists to focus on high-value clinical correlation (31).

### 4.4 Contribution to existing knowledge and addressing research gaps

This study contributes to the emerging body of literature on AI in healthcare by providing evidence at the organizational performance level rather than focusing exclusively on technical algorithm performance or user perceptions. By linking AI-related constructs to measurable operational and clinical outcomes, the findings extend current understanding of how digital health technologies influence system-level performance indicators.

Additionally, the study addresses a temporal gap in the literature by examining AI implementation during routine healthcare operations rather than during emergency or crisis conditions. Many earlier investigations were conducted in response to pandemic-related pressures, emphasizing rapid deployment and triage support. The present findings demonstrate that AI technologies continue to provide measurable benefits under stable operating conditions, indicating their relevance as long-term components of healthcare infrastructure rather than short-term emergency solutions.

### 4.5 Theoretical and managerial implications

From a theoretical perspective, our extension of the Technology Acceptance Model (TAM) is supported by recent integrations of the Task-Technology Fit (TTF) theory in clinical settings. Current research emphasizes that technology characteristics, such as PACS compatibility, system responsiveness, and workflow integration, are essential predictors of AI adoption among medical professionals, validating our assertion that operational usefulness drives technology acceptance (32–35).

From a managerial perspective, the results suggest that healthcare administrators should prioritize integrated AI solutions that align with existing clinical information systems, including Picture Archiving and Communication Systems (PACS) and electronic health records. Successful implementation depends not only on algorithm accuracy but also on system interoperability, user interface design, and workflow compatibility. Strategic investment decisions should therefore consider the broader operational ecosystem in which AI technologies are deployed.

### 4.6 Limitations and future research directions

Despite the relatively large sample size (*N* = 400), this study has some limitations that should be considered when interpreting the findings. First, the cross-sectional design limits the ability to establish causal relationships or assess long-term effects of AI adoption. Longitudinal research designs would enable examination of how performance outcomes evolve over time as technologies mature and user familiarity increases. In addition, the use of purposive non-probability sampling may have introduced selection bias, as healthcare professionals with greater awareness of or interest in AI technologies may have been more likely to participate in the study. Consequently, the findings may not fully represent the perceptions of all imaging personnel across the region.

Second, the study focused primarily on the provider perspective, emphasizing organizational and workforce outcomes. Future research should incorporate patient-centered indicators, including patient satisfaction, trust in AI-assisted diagnoses, and perceived quality of care. The integration of patient-reported outcome measures (PROMs) with operational performance metrics would provide a more comprehensive assessment of AI’s impact on healthcare delivery.

Another important limitation is that the study relied predominantly on self-reported perceptions of AI impact rather than objective departmental performance indicators. Variables such as diagnostic accuracy, operational efficiency, and staff performance were evaluated through participant responses and perceptions rather than direct measurements of reporting turnaround time, workflow throughput, diagnostic error rates, patient waiting time, or imaging productivity metrics. Future studies should incorporate objective operational and clinical indicators to provide a more comprehensive assessment of AI integration outcomes in healthcare systems.

Finally, additional studies across multiple healthcare regions and facility types would strengthen generalizability and support evidence-based policy development related to digital health implementation in diverse healthcare settings.

## 5 Conclusion

This study demonstrates that Artificial Intelligence (AI) integration in medical imaging departments improves organizational performance by enhancing operational efficiency and diagnostic accuracy. AI functions as a strategic enabler of healthcare modernization, supporting service accessibility, resource optimization, and workforce sustainability in resource-constrained settings.

Effective implementation depends on a human–technology partnership in which AI augments clinical expertise, improving decision-making while reducing burnout. These findings provide actionable insights for scaling workflow-integrated AI solutions across diverse healthcare systems. Future research should adopt longitudinal and multi-center approaches to evaluate long-term impacts on patient outcomes and population health.

## Data Availability

The original contributions presented in the study are included in the article/supplementary material, further inquiries can be directed to the corresponding author.
